# Poor sleep quality and associated factors among HIV-positive pregnant women in Northwest, Ethiopia: a facility-based, cross-sectional study

**DOI:** 10.1186/s12888-022-04209-2

**Published:** 2022-08-19

**Authors:** Getasew Legas, Getnet Mihretie Beyene, Sintayehu Asnakew, Amsalu Belete, Tigabu Desie

**Affiliations:** 1 Department of Psychiatry, College of Medicine and Health Sciences, Debre Tabor University, Debre Tabor, Ethiopia; 2 Department of Pediatrics and Child Health Nursing, College of Health Sciences, Debre Tabor University, Debre Tabor, Ethiopia

**Keywords:** Poor sleep quality, HIV-positive, Northwest, Ethiopia

## Abstract

**Background:**

Poor sleep quality during pregnancy might have an impact on adverse birth outcomes like premature rupture of membrane, preterm birth, lifelong neurocognitive impairment, low birth weight, and increased the risk of neonatal morbidity and mortality. In Ethiopia, the magnitude of poor sleep quality among this group of people is extremely limited. So, this study aims to determine the magnitude of poor sleep quality and its associated factors among HIV-positive pregnant women attending public hospitals in Northwest Ethiopia.

**Methods:**

An institution-based cross-sectional study was done using a simple random sampling technique to recruit 411 HIV-positive pregnant women from January to March; 2021. Sleep quality over the last 1 month was measured using the Pittsburgh Sleep Quality Index (PSQI). General anxiety disorder (GAD-7), Sleep Hygiene Index (SHI), and List of Threatening of Experiences (LTE) instruments were used to identify factors associated with poor sleep quality. Bivariate and multivariable logistic regression with odds ratio and 95% CI were employed to identify determinant factors of poor sleep quality. Statistical significance association was declared at *P*-value < 0.05.

**Results:**

A total of 411 out of 423 HIV-positive pregnant women were interviewed, with a response rate of 97.1%. The overall magnitude of poor sleep quality among HIV-positive pregnant was found to be 39.4% with a 95% of confidence interval (CI) (34.3, 44.3). Stressful life events, [AOR = 3.10, 95% CI (1.60, 6.01)], having comorbid general anxiety symptoms [AOR = 2.46, 95% CI (1.58, 3.81)], unplanned pregnancy [AOR = 2.18, 95% CI (1.20, 3.96)], and poor sleep hygiene practice [AOR = 2.23, 95% CI (1.21, 4.10)] were significantly associated with poor quality of sleep.

**Conclusion:**

The overall magnitude of poor sleep quality among HIV-positive pregnant women was high. Stressful life events, poor sleep hygiene, unplanned pregnancy, and comorbid general anxiety symptoms were the determinant factors of poor sleep quality that should be taken high consideration for early detection and appropriate intervention for poor sleep quality in HIV-positive pregnant women.

## Background

Sleep quality is defined as an individual’s satisfaction of sleep with different aspects of the sleep experience including sleep efficiency, sleep latency, sleep duration, and wake after sleep onset [1]. Sleep is a universal behavior, occupying one-third of human life and a significant contributor to energy restoration for proper functioning. Prolonged sleep deprivation might be lead to severe physical illness, the development of mental illnesses, substance use disorders, and cognitive impairment [2]. Globally, over 36.9 million people were living with human immunodeficiency virus/Acquired immunodeficiency syndrome (HIV/AIDS) and sub-Saharan countries are the most severely affected region in Africa estimated at one in every 25 people living with HIV/AIDS [3, 4].

According to the reports of different studies, 50 to 70 million people were suffering from sleep disturbance in the United States [5]. Poor quality of sleep is one of the most common sleep disturbances in HIV/AIDS patients, it accounts for 40–70% of individuals suffering from sleep disturbance compared with the general population [6–8]. Poor sleep quality is a common problem in HIV-positive individuals due to direct effect of the virus and opportunistic infection of the central nervous system [9–11].

Sleep disturbance is also common in pregnant women; approximately, up to 78% of women have complained of different forms of sleep disturbance during their pregnancy [12]. It is a major public health problem both in developed and developing countries, and poor sleep quality during pregnancy is a significant contributor to adverse birth outcomes, growth restriction of the fetus [13], including premature rupture of membrane [14], low birth weight, pre-eclampsia [15], prolonged labor [16], stillbirth [17], abruption of placenta [18], gestational diabetes mellitus [19], gestational hypertension [20], preterm birth [21–24], postpartum depression [25–27], and decreased progesterone level [28].

Studies are carried out to assess the prevalence of poor sleep quality among pregnant women varied across the world. A cross-sectional survey data in China showed that 15.2% of pregnant women had complained of problems related to sleep quality [29]. The other systematic and meta-analysis data in this country also showed the pooled prevalence of poor sleep quality among pregnant women was 54.2% [30]. A study conducted in Bangladesh reported that the magnitude of sleep disturbance was 38.8% [31]. In the USA, 53–71% of pregnant women suffered from poor sleep quality [32], in Vietnam 41.2% [33], in Finland 15% [34], in Malaysia 69.4% [35], in Nepal 28.2% [36], in Taiwan 60% [37], and in Peru 17% [38].

Various factors were identified as a factor of poor sleep quality in the previous studies conducted across the globe, history of abortion, comorbid depression and anxiety [39–42], gestational age (second and third trimester) [43], history of stillbirth, multigravida [44, 45], individual-level of stress [44], stressful life events [41, 46, 47], living in rural areas [48], poor sleep hygiene [42], absence of postnatal care, current use tobacco and khat [49] were the determinant factors of poor sleep quality during pregnancy.

In Ethiopia, the magnitudes of poor sleep quality in pregnant women range from 30.8 to 68.4% [43–45]. Where poor sleep quality is high in low-and-middle-income countries including Ethiopia. But limited studies were conducted to assess the magnitude of poor sleep quality among HIV-positive pregnant women in Ethiopia. Therefore, assessing the magnitude and associated factors of poor sleep quality among these groups of women is important for early identification, and intervention; to reduce the effect of poor sleep quality on pregnancy and adverse birth outcomes.

## Methods

### Study design and period

An institution-based cross-sectional study was conducted to determine the magnitude and associated factors of poor sleep quality among HIV-positive pregnant women in Northwest Ethiopia from January to March 2021.

### Study area

The study was conducted in the Amhara region selected referral hospitals. The capital city of the Amhara region is Bahirdar which is located northwest part of Ethiopia. The city is the seat of the regional government and is 564 km far from the capital city of Ethiopia (Addis Ababa). The Amhara region is divided into 13 zones named West Gojjam, Awi Zone, North Shewa, Central Gondar, South Gondar, North Gondar, East Gondar, East Gojjam, Oromia zone, Waghimra, South Wollo, North Wollo, and Bahirdar (special zone). According to the recent population census report; the total population size of the Amhara region is estimated at 17,221,976. Of these, 12.27% of the population is residing in the urban area.

### Source population

All HIV-positive pregnant women whose age was 18 years and above come for PMTCT (prevention of mothers-to-child-transmission) follow-up in selected Amhara region referral hospitals, Northwest, Ethiopia.

### Study population

All HIV-positive pregnant women whose age was 18 years and above come for PMTCT follow-up in selected Amhara region referral hospitals during the study period.

### Sampling procedure and sample size determination

A single population proportion formula was used to determine the sample size by taking the prevalence of poor sleep quality among HIV-positive pregnant women was 50% (due to the reason of unknown prevalence) with a 5% margin of error, and 95% confidence with a 10% non-response rate; the final sample size was 423.

The study subjects were recruited with a simple random sampling technique. The selected referral hospitals in the Amhara region were Woldia, Debre Tabor, Gondar, and Felege Hiwot. In each of the selected hospitals, the number of study participants was obtained from health care providers working in the prevention of mothers-to-child-transmission (PMTCT) clinic. The list of study subjects from each hospital was used as a sampling frame. Then, each study participant was selected by the lottery method.

### Inclusion and exclusion criteria

All HIV-positive pregnant women whose age was 18 years and above come for PMTCT follow-up in selected Amhara region referral hospitals were included in the study during the data collection period. HIV-positive pregnant women who were unable to communicate due to severe illness were excluded from the study.

### Study variables

#### Dependent variable

**Poor sleep quality** (1 = yes, No = 0).

#### Independent variables

##### Socio-demographic variables

Age, ethnicity, educational status, marital status, and residence.

##### Clinical factors

family history of mental illness, history of stillbirth, sleep hygiene practice, and comorbid general anxiety symptoms.

##### Psychosocial factors

stressful life events.

#### Operational definition

##### Sleep quality

measured using the Pittsburgh sleep quality index (PSQI) at a cut-off point greater than 5 considered poor sleep quality [50].

##### Anxiety

assessed using GAD-7, a score of ≥ 10 having general anxiety symptoms [51].

##### Poor sleep hygiene

measured using a sleep hygiene index (SHI), a score above the mean on the scale was categorized as poor sleep hygiene practice [52].

##### Stressful life events

The presence of stressful life events in the last 6 months was assessed by using a 12-item List of Threatening Experiences (LTE). A study participant having one or more stressful life events is considered as having stressful life events [53].

##### Family history of mental illness

was assessed by asking a semi-structured question “Did you have a family history of mental illness?” with a positive response of “Yes” categorized by having a family history of mental illness.

##### History of stillbirth

was measured by asking “Have you had a history of stillbirth?” with a positive response of “yes” considering having a previous history of stillbirth.

### Data collection procedures and instruments

A semi-structured interviewer-administered questionnaire was used that contains socio-demographic factors, psychosocial factors (stressful life events, history of stillbirth, and unplanned pregnancy) clinical factors (trimester, family history of mental illness, and comorbid general anxiety symptoms).

Sleep quality was measured using a 19-item of Pittsburgh Sleep Quality Index (PSQI). The tool is categorized into seven components containing subjective sleep quality, sleep latency, sleep duration, habitual sleep efficiency, sleep disturbances, daytime dysfunction, and use of sleep medications. Each item of PSQI components has been rated on a three-point scale (0–3), ranging from 0 to 21. A total score of 5 or more according to the PSQI questionnaire was considered as having poor sleep quality over the last month. The tool was validated in Ethiopia with sensitivity and specificity results of 89.6% and 86.5% respectively [50].

General anxiety symptoms in the last 2 weeks were measured using the General Anxiety Disorder questionnaire (GAD-7). The instrument has seven items and each item of questions is rated on a three-point scale, 0 (not at all), 1 (Several days), 2 (More than half the days), and 3 (Nearly every day) with a total score of 0–21. A score of five indicates mild anxiety symptoms, ten indicates moderate anxiety symptoms and fifteen indicates severe symptoms of anxiety [51].

The presence of stressful life events in the last 6 months was assessed by using a 12-item List of Threatening Experiences (LTE). Each item of the instrument has been rated on a two-point scale (yes/no), “1” and “0” indicates the presence and absence of individual level of stress over the last 6 months respectively [53].

Sleep hygiene was measured by a 13-item of sleep hygiene index (SHI). The tool measured the practice of sleep hygiene behavior and each item SHI instrument was rated on a five-point Likert scale characterized by 0(never), 1(rarely), 2(sometimes), 3(frequent), and 4(always) with a total score of 0 to 52. A scoring of above the mean indicates poor sleep hygiene [52].

To examine a family history of mental illness, study subjects were asked: “Did you have a family history of mental illness?” with a response of “yes” having a family history of mental illness. To assess the previous history of stillbirth, study subjects were asked: “Have you had a history of stillbirth?” with a response of “yes” considered a previous history of stillbirth.

### Data quality control issues

The data was collected by four trained BSc holder nurses and supervised by trained psychiatry professionals after 2 days of training. The data quality was ensured by giving training on the data collection instrument, sampling procedure, and regular supervision. The questionnaire was also translated from English to the Amharic language (local working language).

### Data processing and analysis

The collected data were entered into Epi-data 3.1 and then exported to SPSS - version 21 for analysis. The bivariate logistic analysis was applied to select determinant factors. All independent variables with a *p*-value < 0.25 in the bivariate analysis were a candidate to enter the multivariable logistic regression model to control the confounding effect. Multivariable logistic regression was used to identify factors associated with poor sleep quality and *p*-value < 0.05 with 95% of CI considered statistically significant.

## Results

A total of 411 out of 423 HIV-positive pregnant women were interviewed, with a response rate of 97.1%. The majority of respondents 380 (92.5%) were Amhara. Half of the study subjects, 206 (50.1%) were in the age group of 18–30 years; regarding religion, almost three-fourth of study participants, 284 (69.1%) were orthodox Christian followers; 363 (86.6%) were currently married, Over half study the respondents, 236 (57.4%) of respondents were urban areas residents, and 255(62%) of respondents were married (Table 1).

**Table 1:** Socio-demographic characteristics of respondents in Northwest Ethiopia, 2021

Variable	Category	Frequency	Percentage
Age	18–30	206	50.1%
	31–45	205	49.9%
Ethnicity	Amhara	380	92.5%
	other	31	7.5%
Educational status	Unable to read and write	95	23.1%
	1–8 grade	127	30.9%
	9–12 grade	63	15.3%
	Diploma & above	126	30.7%
Religion	Orthodox	284	69.1%
	Others	127	30.9%
Marital status	Currently married	355	86.4%
	Currently not married	56	13.6%
Residence	Rural	175	42.6%
	Urban	236	57.4%

### Clinical and psychosocial factors of HIV-positive pregnant women

Of the total study subjects, 359 (87.3%) respondents hadn’t had stressful life events, and three hundred sixty-nine, (89.8%) hadn’t a history of stillbirth. Regarding comorbid mental illness, 268 (65.2%) of respondents had no comorbid anxiety symptoms, 379 (92.2) of study subjects hadn’t a family history of mental illness, and over three-fourth of study participants, 333(81.0%) had good sleep hygiene practice (Table 2).

**Table 2:** Distribution of clinical and psychosocial factors of respondents in Northwest, Ethiopia, 2021

Variable	Category	Frequency	Percentage
Unplanned pregnancy	yes	63	15.3
	no	348	84.7
Co-morbid anxiety	yes	143	34.8
	no	268	65.2
History of still birth	Yes	42	10.2
	No	369	89.8
Stressful life events	Yes	52	12.7
	No	359	87.3
Sleep hygiene	good	333	81
	Poor	78	19
Trimester	I	94	22.9
	II	143	34.8
	III	174	42.3
Family history of mental illness	Yes	32	7.8
	No	379	92.2

### The magnitudes of poor sleep quality

The overall magnitude of poor sleep quality among HIV-positive pregnant was found to be 39.4% with a 95% of confidence interval (CI) (34.3, 44.3).

### Factors associated with poor sleep quality in HIV-positive pregnant women

In this study, poor sleep hygiene, history of stillbirth, unplanned pregnancies, stressful life events, and comorbid anxiety were significantly associated with poor sleep quality on bivariate analysis. In the final adjusted multivariable analysis, comorbid general anxiety symptoms, poor sleep hygiene, stressful life events, and unplanned pregnancies were found to be significantly associated with poor sleep quality.

The odds of developing poor sleep quality among HIV-positive pregnant women who had unplanned pregnancies were 2-times higher compared with individuals who had planned pregnancies [AOR = 2.18, 95% CI (1.20, 3.96)]. Study subjects who had Stressful life events were 3 times more likely to have poor sleep quality than women who hadn’t a stressful life event [AOR = 3.10, 95% CI (1.60, 6.01)]. The odds of developing poor sleep quality among pregnant women who had poor sleep hygiene was 2-fold times higher than women who had good sleep hygiene [AOR = 2.23, 95% CI (1.21, 4.10)]. The likelihood of developing poor sleep quality was 2.46 times higher among study participants who had comorbid general anxiety symptoms compared with those who hadn’t comorbid general anxiety symptoms [AOR = 2.46, 95% CI (1.58, 3.81)] (Table 3).

**Table 3:** Bivariate and Multivariable analyses of poor sleep quality among HIV positive pregnant women in Northwest Ethiopia, 2021

Variables	Category	Sleep quality		COR (95% CI)	AOR (95% CI)
		**Good**	**Poor**		
Unplanned pregnancy	No	223	125	1	1
	Yes	26	37	2.53(1.46, 4.38)	2.18 ((1.20, 3.96))*
Comorbid anxiety	No	182	86	1	1
	Yes	67	76	2.40(1.58, 3.64)	2.46 (1.58, 3.81)*
Sleep hygiene	Good	216	117	1	1
	Poor	33	45	2.51(1.53, 4.16)	2.23 (1.21, 4.10)*
Stressful life events	No	232	127	1	1
	Yes	17	35	3.76(2.02 6.98)	3.10 (1.60, 6.01)*
History of still birth	No	231	138	1	1
	Yes	18	24	2.23(1.16, 4.26)	1.32(0.60, 2.87)

*COR *Crude Odds Ratio, *AOR *Adjusted Odds Ratio, *P < 0.05

## Discussion

As per our knowledge, this is the first study to assess poor sleep quality among HIV-positive pregnant women in Africa. In this study, the overall magnitude of poor sleep quality among HIV-positive pregnant was found to be 39.4% with a 95% of confidence interval (CI) (34.3, 44.3). The magnitude of poor sleep quality in the current study was consistent with a study conducted in Bangladesh at 38.8% [31], and Vietnam 41.2% [33]. However, the magnitude of poor sleep quality in the current study is lower than studies reported in Malaysia 69.4% [35], in Taiwan 60% [37], in the USA 53 to 71% [32], in Northern Ethiopia 68.4% [43], and a systematic and meta-analysis data in China which was 54.2% [30].

Conversely, the current study was higher than the estimated magnitude of poor sleeping quality in China 15.2% [29] and 34.14% [14], in Finland 15% [34], and in Southwest Ethiopia 30.8% [44]. The possible reason for the discrepancy might be variations in instrumental used to measure the quality of sleep in HIV-positive pregnant women and variations in the number of sample sizes, cultural, and economic differences might have a significant contribution to this discrepancy.

In the present study, the odds of developing poor sleep quality were 3 times higher among HIV-positive pregnant women who had stressful life events compared with individuals who hadn’t stressful life events. This finding was in line with studies done in China [46, 47]. This is explained by the fact that stressful life events had their own impact on the maladaptive hypothalamic-pituitary-adrenal (HPA) system [54]. Negative life events might lead to greater activation of the locus coeruleus norepinephrine system and increased HPA axis, which may also increase excitement and precipitate poor quality of sleep [55].

HIV-positive pregnant women who had unplanned pregnancies were 2-times more likely to have poor sleep quality compared with pregnant women who had planned pregnancies. But, the finding of this study has no similar literature that showed unplanned pregnancies is significantly associated with poor sleep quality. The possible reason might be that HIV-positive pregnant women who had unplanned pregnancy might have psychological and emotional distress. Due to this reason, women who had unplanned pregnancies might have excessive worries. As a result of this, unplanned pregnancies might be a risk factor for poor sleep quality.

In the present study, participants with poor sleep hygiene practice were more likely to have a poor quality of sleep when compared with participants with good sleep hygiene practice. The significant association between poor sleep hygiene practice and poor sleep quality in the present study is similar to previous Ethiopian studies [42, 49] and a study done in Nigeria [56]. The possible explanation might be the risk of poor sleep quality in study subjects who had poor sleep hygiene practice might be justified by poor knowledge regarding the importance of sleep hygiene practice leading to poor sleep quality [57, 58].

Finally, we found an association between comorbid general anxiety symptoms and poor sleep quality. This is probably due to the fact that individuals, who had general anxiety symptoms, have an impact on poor quality of life and lead to poor sleep quality [59]. The finding is similar to a study done in Ethiopia [41] and the US [60].

### Limitation of the study

This study didn’t show the cause and effect relationship of the dependent and determinant variables. The other limitations might be interviewer bias and recall problems. Finally, we have not assessed factors like chronic physical illness, perceived stigma due to HIV/AIDS, depression, HIV- status of the previous child, current use of substances (alcohol, Khat, and tobacco), and other comorbid mental illnesses that may have higher significance for poor sleep quality.

## Conclusion

The overall magnitude of poor sleep quality among HIV-positive pregnant women was high. Stressful life events; poor sleep hygiene practice, unplanned pregnancy, and comorbid general anxiety symptoms were the determinant factors of poor sleep quality. Therefore; we recommend that health care providers working in PMTCT clinics focused on early regular screening for sleep disturbance, and give higher attention to HIV-positive pregnant women who had an unplanned pregnancy, poor sleep hygiene practice, stressful life events, and comorbid general anxiety symptoms. Strengthening the referral linkage between health care providers working in PMTCT clinics and mental health specialists should be promoted.

## Data Availability

all data are available in the manuscript.

## Abbreviations

PSQI: Pittsburgh Sleep Quality Index
GAD-7: General anxiety disorder (GAD-7)
SHI: Sleep Hygiene Index
PMTCT: Prevention of mothers-to-child-transmission
HIV: Human immune deficiency virus
AIDS: Acquired Immune Deficiency Syndrome
LTE: List of Threatening Experiences
